# The Mapping of Predicted Triplex DNA:RNA in the *Drosophila* Genome Reveals a Prominent Location in Development- and Morphogenesis-Related Genes

**DOI:** 10.1534/g3.117.042911

**Published:** 2017-05-17

**Authors:** Claude Pasquier, Sandra Agnel, Alain Robichon

**Affiliations:** * Université Côte d'Azur, CNRS, I3S, France; † Université Côte d'Azur, INRA, CNRS, ISA, France

**Keywords:** Hoogsteen rules, development, epigenetics, gene networks, triplex DNA:RNA

## Abstract

Double-stranded DNA is able to form triple-helical structures by accommodating a third nucleotide strand. A nucleic acid triplex occurs according to Hoogsteen rules that predict the stability and affinity of the third strand bound to the Watson–Crick duplex. The “triplex-forming oligonucleotide” (TFO) can be a short sequence of RNA that binds to the major groove of the targeted duplex only when this duplex presents a sequence of purine or pyrimidine bases in one of the DNA strands. Many nuclear proteins are known to bind triplex DNA or DNA:RNA, but their biological functions are unexplored. We identified sequences that are capable of engaging as the “triplex-forming oligonucleotide” in both the pre-lncRNA and pre-mRNA collections of *Drosophila melanogaster*. These motifs were matched against the *Drosophila* genome in order to identify putative sequences of triplex formation in intergenic regions, promoters, and introns/exons. Most of the identified TFOs appear to be located in the intronic region of the analyzed genes. Computational prediction of the most targeted genes by TFOs originating from pre-lncRNAs and pre-mRNAs revealed that they are restrictively associated with development- and morphogenesis-related gene networks. The refined analysis by Gene Ontology enrichment demonstrates that some individual TFOs present genome-wide scale matches that are located in numerous genes and regulatory sequences. The triplex DNA:RNA computational mapping at the genome-wide scale suggests broad interference in the regulatory process of the gene networks orchestrated by TFO RNAs acting in association simultaneously at multiple sites.

Noncanonical DNA structures such as the triplex have been the subject of extensive studies in biophysics using simple synthetic templates. These structures may also involve RNA as a third strand to achieve tertiary structures (Cheng and Pettitt 1992; Escudé *et al.* 1993; Frank-Kamenetskii and Mirkin 1995; Morgan and Wells 1968; Plum 1997; Roberts and Crothers 1992, 1996; Sun *et al.* 1996). These combinatorial assemblages have been confirmed by thermodynamic analyses that enabled a determination of the constants of association and dissociation of the third strand to double-stranded DNA depending on pH and its length (Cheng and Pettitt 1992; Escudé *et al.* 1993; Frank-Kamenetskii and Mirkin 1995; Morgan and Wells 1968; Plum 1997; Roberts and Crothers 1992, 1996; Sun *et al.* 1996). Although the measured affinities were able to indicate the stability of triplexes, their presence and biological functions *in vivo* have been poorly explored due to technical limitations. However, we have solid and indirect evidence of their putative roles and existence from the finding that a few transcription factors and other DNA-binding proteins show high-affinity binding to some triplexes (Buske *et al.* 2011). Their roles in the genomic context of regulation of gene networks remain elusive.

Basically, strict sequence alignments are required for stable triple-helix formation via hydrogen bonds between the bases of a DNA duplex and a third RNA strand according to Hoogsteen base-pairing rules. The sequences in the DNA duplex must show a succession of pyrimidine bases [T, C] in one strand and a consequent succession of purine base repeats [G or A] in the opposite strand. On the RNA side, the sequences composed of pyrimidine bases [U, C] must be the copy of one DNA strand and bind parallel to the DNA duplex, or the sequences of the purine bases [G, A] must be the copy of one DNA strand and bind antiparallel to the DNA duplex. A third possibility is the sequence of purine-pyrimidine bases [G, T, or A] in the RNA strand that binds antiparallel and in which T or A can indistinctly bind to the A:T pair in the DNA strand (Cheng and Pettitt 1992; Escudé *et al.* 1993; Frank-Kamenetskii and Mirkin 1995; Morgan and Wells 1968; Plum 1997; Roberts and Crothers 1992, 1996; Sun *et al.* 1996). The minimal requirement for the third strand to form a DNA:RNA triplex is a length of nine bases; the strand is typically 10–30 nt in length (Buske *et al.* 2011; Cheng and Pettitt 1992; Escudé *et al.* 1993; Frank-Kamenetskii and Mirkin 1995; Knauert and Glazer 2001; Morgan and Wells 1968; Plum 1997; Roberts and Crothers 1992, 1996; Sun *et al.* 1996). However, the affinity between the strands when the matches are perfect increases with the length of the third strand. The large number of compatible sequences over 20 bases in length in the RNA presenting the potential to form triplexes in the genome strongly suggests that these structures might have a large spectrum of biological functions under their control, which are unknown to date.

We used Triplexator (Buske *et al.* 2011) to investigate the putative triple helix according to the Hoosgteen rules, involving a double-stranded nucleic acid and a single-stranded RNA on a genome-wide scale and hypothesizing that these structures might constitute a new component of epigenetic regulation in gene imprinting, developmental biology, and environmental adaptation. The melting curves in biophysics studies have led to the general acceptance that a higher affinity is obtained when the third strand is RNA instead of DNA (Cheng and Pettitt 1992; Escudé *et al.* 1993; Morgan and Wells 1968; Roberts and Crothers 1992). More importantly, many authors have attempted *in vivo* investigations in diverse organisms to determine the roles of such structures in relation to chromosome architecture, gene regulation, and human pathologies, such as cancer or hereditary neurological disorders (Buske *et al.* 2011). Furthermore, the aim of investigating the putative extent of these structures in genomes was inspired by the fact that we found that a large panel of nuclear proteins have been reported to bind triplex structures. For instance, the high mobility group proteins HMG1 and HMG2 are chromatin-associated proteins and are able to bind triplexes (Buske *et al.* 2011; Suda *et al.* 1996). Their roles seem to involve the higher architecture of chromosomes and the distribution of euchromatin/heterochromatin (Buske *et al.* 2011; Suda *et al.* 1996). As another example, the GAGA transcription factor is reported to bind a pyrimidine triplex motif with the same affinity and specificity as for a duplex (Jiménez-García *et al.* 1998). These triplex structures inhibit the expression of genes located downstream (Jiménez-García *et al.* 1998). The GAGA DNA motif and GA factor in *Drosophila* are strongly involved in developmental patterning of the body by regulating the homeotic bithorax genes in the Antennapedia complex (Granok *et al.* 1995; Lehmann 2004). Moreover, GA factors are known to participate in the epigenetic maintenance of heterochromatin and the silencing of genes embedded in this structure (Granok *et al.* 1995; Lehmann 2004). As another example, CDP1 (centromere binding factor) binds to a purine motif triplex with high affinity (Kd 5 nM), whereas the pyrimidine motif triplex along with the duplex shows a low affinity (5 µM) (Musso *et al.* 2000). CDP1 mutants show defects in chromosome segregation during mitosis and a high level of chromosome fragmentation (Musso *et al.* 2000). Moreover, the dihydrofolate reductase (DHFR) promoter is able to engage the triplex structure with some RNAs, which prevents transcription factor binding (Buske *et al.* 2011; Martianov *et al.* 2007). Finally, on a health-related topic, Friedreich ataxia is caused by the insertion of GAA/TTC repeats located in the first intron of the FXN gene, creating triplex structures (Soragni *et al.* 2008). The histones surrounding the ataxia triplex structures show hypoacetylation and hyper-methylation (Soragni *et al.* 2008). Altogether, these examples highlight that pieces of RNA presenting sequences that are compatible with Hoogsteen rules might have a widespread role in epigenetically regulating a large number of genes.

We chose to work on the simple genetic model *Drosophila melanogaster* because of the availability of genome annotation information with substantial descriptions of mutant phenotypes in multiple databases. We investigated whether the two classes of RNA (lncRNA and mRNA) sheltered compatible sequences to engage the triplex structure with DNA, raising the idea that they might have a widespread role in chromosome 3D architecture and gene regulation. lncRNAs are known to specifically and restrictively recruit promoters, resulting in downregulation or overexpression of targets genes (Mercer *et al.* 2009; Rinn and Chang 2012). Many lncRNAs are composed of a few hairpin structures that form higher order spatial architecture by their association with hairpin-bound proteins. Each hairpin motif is supposed to bind tightly to a specific nuclear protein involved in the transcription machinery and 3D chromatin organization (Chen 2016; Goff and Rinn 2015; Li *et al.* 2016; Quinn and Chang 2015; Rinn and Chang 2012; Tsai *et al.* 2010). These multimeric associations form extensive networks of ribonucleoprotein (RNP) complexes that target genomic locations (Chen 2016; Goff and Rinn 2015; Li *et al.* 2016; Quinn and Chang 2015; Rinn and Chang 2012; Tsai *et al.* 2010). Furthermore, lncRNAs appear to produce alternative splice variants acting as modular scaffolds for variable RNP complexes that specify chromatin states (Chen 2016; Goff and Rinn 2015; Li *et al.* 2016; Mercer *et al.* 2009; Quinn and Chang 2015; Rinn and Chang 2012; Tsai *et al.* 2010). lncRNAs are noncoding and defined as being larger than 200 nt in length (Chen 2016; Goff and Rinn 2015; Quinn and Chang 2015; Rinn and Chang 2012). Despite these advances, most lncRNAs remain partially or not at all characterized. A large fraction of these lncRNAs are predominantly or strictly localized to the nucleus, although a few seem exclusively present in the cytoplasm (Cabili *et al.* 2015; Chen 2016; Geisler and Coller 2013).

While many lncRNAs are rapidly degraded after or during the completion of their transcription, a significant fraction of the lncRNAs are stable nuclear transcripts that are 5′-capped, spliced, and polyadenylated similar to mRNAs (Chen 2016; Goff and Rinn 2015; Li *et al.* 2016; Quinn and Chang 2015; Rinn and Chang 2012; Wilusz *et al.* 2009). While all these structural modifications are known to stimulate the nuclear export of protein-coding transcripts, this is not the case for lncRNAs, most of which remain surprisingly locked inside the nuclear perimeter (Cabili *et al.* 2015; Chen 2016; Geisler and Coller 2013).

Because some lncRNAs are located inside the introns of genes, this finding raises the possibility that pieces of intronic RNA in the mRNA collection may have widespread regulatory functions beyond translation. The TFOs (triplex-forming oligonucleotides) that were found in the RNA (pre-lncRNA and pre-mRNA) were blasted against the entire genome, and then, location analysis was performed. The genes to which the TFO matched were submitted to wide-scale genome GO enrichment analysis in order to find networks of genetic interactions. The results unambiguously suggest that genes involved in development and morphogenesis might be a preferential target for TFO binding.

## Materials and Methods

### Data gathering

Data for the *D. melanogaster* genome are stored in the FTP directory of the FlyBase website that stores FASTA sequences. We downloaded FASTA sequences of genes, introns, exons, transcripts (mRNAs and lncRNAs), and chromosomes. Our work was based on version 6.06 of the data, released on June 26, 2015.

### Triplex identification

There are several algorithms dedicated to the identification of triplexes. Triplexator (Buske *et al.* 2012) is the first computational framework that integrates all aspects of triplex formation. It allows the detection of triplex-forming oligonucleotides (TFOs), triplex-target sites (TTSs), and the localization of TFO:TSS matchings that are potential sites for triplex formation. LongTarget (He *et al.* 2015) includes similar features plus the ability to determine the best TFO:TTS matchings and to evaluate triplex stability. Unfortunately, the software is no longer available for download. Trident (Paugh *et al.* 2016) is dedicated to the identification of microRNAs favoring triplex formation. The tool cannot be used to detect TFOs in long RNA sequences. In our study, we used Triplexator, which has all the features that we needed.

The FASTA sequences of genes encoding lncRNAs and mRNAs were processed to identify TFOs of a minimum length of 20 bases that complied strictly to the triplex formation rules. The following motifs were searched:

the pyrimidine motif, [TC], where thymines and cytosines bind parallel;
the purine motif, [GA], where guanines and adenines bind antiparallel; and
the purine-pyrimidine motif, [GT], where guanines and thymines bind either parallel or antiparallel with respect to the purines in the duplex.

This search led to the identification of 896 TFOs in pre-lncRNAs and 12,898 TFOs in pre-mRNAs.

Using the files available in FlyBase, we distinguished the sequences corresponding to the introns and the sequences corresponding to the exons. We show the number of TFOs corresponding to each of these categories in Table 1.

**Table 1:** Distribution of TFOs

	lncRNA		mRNA	
	Number	%	Number	%
Genes	896	100.00	12,898	100.00
Introns	626	69.87	9,024	70.00
Exons	270	70.13	3,874	30.00

Details of the localization of TFOs found in pre-RNAs are presented in columns “lncRNA” and “mRNA”. The distribution of TFOs between introns and exons is very similar for the two kinds of RNAs: 70% of TFOs are located in introns.

The program Triplexator was also used to process the FASTA sequences of the chromosomes, genes, and exons in order to identify TTSs and to detail their distribution. For this search, no mismatches were allowed, and a minimum length of 20 bases was also used. Using these parameters, the program identified 9702 target sites on the chromosomes.

Potential triplex sites were also computed with the Triplexator program using perfect matches between TFOs and TTSs and a minimum length of 20 bases. Normally, each TFO was potentially able to form a triplex with the TSS located at the same position on the complementary strand. These systematic auto-matches did not bring anything to the study of the importance of the TFO-TSS matches on a genome-wide scale. We decided not to take into account these self matches. Accordingly, approximately one quarter of the TFOs, whose only matches are with the TSSs located at the same position on the reverse strand, were removed. However, most of the time, each TFO could target TTSs at numerous sites, including matches that differed in a single nucleotide (this could be due to a simple shift or because the region suitable for triplex formation included one of a smaller size). The analysis of raw outputs generated by the program has led to a count of 2,249,658 potential triplexes with TFOs originating from pre-lncRNAs and 29,339,437 potential triplexes for TFOs originating from pre-mRNAs. We filtered these results by considering that two sites that overlap over >10 bases constituted the same triplex. This processing allowed a reduction in the number of potential triplexes to 354,963 and 4,317,708 for pre-lncRNAs and pre-mRNAs, respectively. We performed the same processing for TFOs originating from introns and TFOs originating from exons.

All statistics are calculated from results obtained by setting the number of maximum mismatches to zero. We used this parameter because allowing some errors increased the computation time and the number of identified sequences. For example, with a maximum of one error every 20 bases (an error rate of 5%), the processing time was multiplied by 35 (12 hr *vs.* 20 min), and the sequences to analyze were much more numerous (3,083,421 triplexes originating from pre-lncRNAs *vs.* 354,963 and >38 million triplexes originating from pre-mRNAs). However, at least for a proportion of the errors that did not exceed 5%, this consideration did not change the overall repartition of patterns, as seen by comparing Supplemental Material, Table S17 in File S4 with Table 2.

**Table 2:** Distribution of TTSs and triplexes on the genome

	TTS		Pre-lncRNA		Pre-mRNA	
	Number	%	Number	%	Number	%
Genome	9702	100.00	354,963	100.00	4,317,708	100.00
Genes	7486	77.16	258,022	76.22	3,290,740	76.21
Introns	5801	59.79	223,837	66.12	2,845,355	65.90
Exons	1685	17.37	34,185	10.10	445,385	10.31

A search for sequences able to accommodate a third strand identified 9702 triplex-target sites along the genome. The column entitled “TSS” details the localization of these sites according to the absolute number and percentage of the total number of TTSs. Details on the localization of the potential triplex sites found in the full genome are presented in columns “pre-lncRNA” and “pre-mRNA” for TFOs originating from pre-lncRNA and pre-mRNA, respectively. The three columns show a very similar distribution: around four fifths of the sites are localized inside genes, with a significant majority of them occurring in introns.

### Estimation of the presence of paired double helixes in TFOs

We searched the RNAs for every sequence of at least four bases that could fold back on itself to form a loop that varied in size from 4 to 8 bases long. In addition, the presence of long-loop regions, such as those occurring in long-range pseudoknots, were identified by searching for the RNA:RNA interactions of at least 10 bases separated by a loop region up to 500 bases. We did a systematic search of the pre-RNAs (for both lncRNAs and mRNAs) and mature forms. The presence of the intermolecular interactions in the mature RNA TFOs was estimated at 15.5 and 16.4% for lncRNAs and mRNAs, respectively. The presence of these potential paired double helices in the pre-RNA TFOs was estimated at 25.7 and 21.9% for pre-lncRNAs and pre-mRNAs, respectively.

### Gene ontology enrichment analysis

The distributions of potential triplexes by genes formed with pre-lncRNA TFOs and pre-mRNA TFOs highlight the fact that a limited number of genes concentrate most of the triplexes (Tables S12 and S13 in File S4 list the genes most targeted by the pre-lncRNA TFOs and pre-mRNA TFOs, respectively). However, the contribution of some genes to the formation of the triplexes was more due to their above-average lengths than to their enrichment in TSS zones. To highlight triplex-enriched genes, we normalized the count of the triplexes by taking into account the lengths of the genes. As a baseline, we used all the matching combinations between TFO and TSS divided by the sum of gene lengths. On average, we could count 32 TFO-TSS matches every 1000 bases (File S1 contains lists of genes targeted by pre-lncRNA TFOs and pre-mRNA TFOs sorted by the density of the TFO-TSS matches). For Gene Ontology enrichment analysis, we used the lists of genes that accounted for 75% of the total number of triplexes and that grouped a count of TFO-TSS matches per 1000 bases above the average. This list represented 35 genes targeted by pre-lncRNA TFOs and 447 genes targeted by pre-mRNA TFOs.

Transcripts that contributed to the formation of TFO-TTS matches had similar distributions (Tables S8 and S9 in File S4 list the pre-lncRNAs and pre-mRNAs that contributed the most to the formation of triplexes). For Gene Ontology enrichment analysis, we used the transcripts that contributed to the formation of >75% of the triplexes with a density of TFO-TSS matches above the average. These transcripts represented 381 pre-mRNAs and 15 pre-lncRNAs (File S1 contains lists of pre-lncRNAs and pre-mRNAs that contributed to the formation of triplexes sorted by the density of the TFO-TSS matches). For genes encoding lncRNAs that had few annotations, no significant enrichment was found.

Gene Ontology enrichment analysis was performed with the online service provided on the Gene Ontology Consortium website: http://geneontology.org/.

### Analysis of genetic interactions

Genetic interactions for *D. melanogaster* were downloaded from the FTP directory of the FlyBase website that stored the precomputed files. Our analysis was based on release “FB2015_03.”

The 35 genes that were most targeted by pre-lncRNA TFOs are included in the list of genes that were most targeted by pre-mRNA TFOs. We used the list of 447 genes enriched in the triplexes to build a network showing the genetic interactions that existed between two different genes on the list. After this filtering, we obtained a list of 76 interacting genes. From this list, 24 genes belonged to disconnected groups of two or three interacting genes. The 52 remaining genes shaped a large network. Network topology was analyzed by Cytoscape (Shannon *et al.* 2003) and the plugin ClusterViz (Wang *et al.* 2014). The EAGLE algorithm (Shen *et al.* 2009) in ClusterViz was used to identify three network modules containing 8–34 genes. The EAGLE clustering method, included in ClusterViz, was able to find spanning clusters, meaning that in our case, some genes were common to several clusters. Gene Ontology enrichment analyses were performed on the list of genes composing these modules and used to annotate the graph presented in Figure 3.

### Isolation of the triplex structures with a biotinylated probe from the cleaved genome

The genomic DNA extraction was performed with the Isolate II Genomic DNA kit from Bioline. *Drosophila* were directly ground in lysis buffer and incubated for 3 hr. Double-stranded genomic DNA fragments were produced using dsDNA Fragmentase from New England Biolabs. The incubation was performed for 20 min in order to obtain 200–300 bp fragments. After the digestion, the DNA was left at room temperature for 1 hr to let the triplex reform. The fragments were incubated with TO biotin (5 µl of TO for 50 µl of DNA) for 30 min at 25°. Then we added TO (without biotin) in excess to compete for the “nonspecific” DNA duplex binding, at a concentration of 100 µg/µl. The incubation was performed for 30 min at 25°. Then the DNA-TO biotin complexes were isolated using a Dynabeads MyOne Streptavidin C1 Kit from Invitrogen. The beads were used in excess (10 µg for 50 µl of DNA). Two washes at room temperature were performed according to the kit instructions. The beads were then placed at 90° for 30 min to break the binding between the streptavidin and biotin, and the supernatant was collected. A measurement by Nanodrop showed a very low amount of DNA. To amplify these few fragments, we used a High-Sensitivity DNA Library Preparation Kit from Abcam. This kit included a DNA end polishing, an adaptor ligation, an amplification by PCR, and a clean-up of the library. The fragments were cloned into the pCR 2.1-TOPO vector using a TOPO TA Cloning Kit from Invitrogen, with an incubation of 30 min. TOP10 chemically competent bacteria were transformed with the resulting plasmids. The bacteria were plated on LB agar petri dishes prepared with kanamycin and X-gal to discriminate bacteria with recombinant plasmids. Approximately 300 colonies were analyzed by Sanger sequencing.

### Data availability

More detailed results (3 figures and 18 tables) are available within File S1, File S2, and File S3. Complete versions of data listed in Tables S8 and S9 in File S4 and Tables S12 and S13 in File S4 are available in File S1 while File S2 contains the complete versions of data listed in Tables S10 and S11 in File S4 and the complete GO annotations used to build Tables S14–S16 in File S4. All data associated with Figure 3 are listed in File S3: list of genes on the graph, composition of each cluster, GO enrichments obtained for the whole graph, and the three identified clusters.

## Results

We analyzed the pre-lncRNA and pre-mRNA sequences of *D. melanogaster* to identify the putative sites of triplex formation for at least 20 ribonucleotides that comply strictly to the triplex formation rules. Of the 2470 pre-lncRNA and the 13919 pre-mRNA sequences stored in FlyBase, a notable proportion of them, between 10 and 20% (269 and 2888, respectively), contain triplex-forming oligonucleotide (TFO) patterns. Most of them include only one TFO, but several transcripts contain more (Table S1 in File S4 lists the transcripts including >50 TFOs).

**Figure 1:**
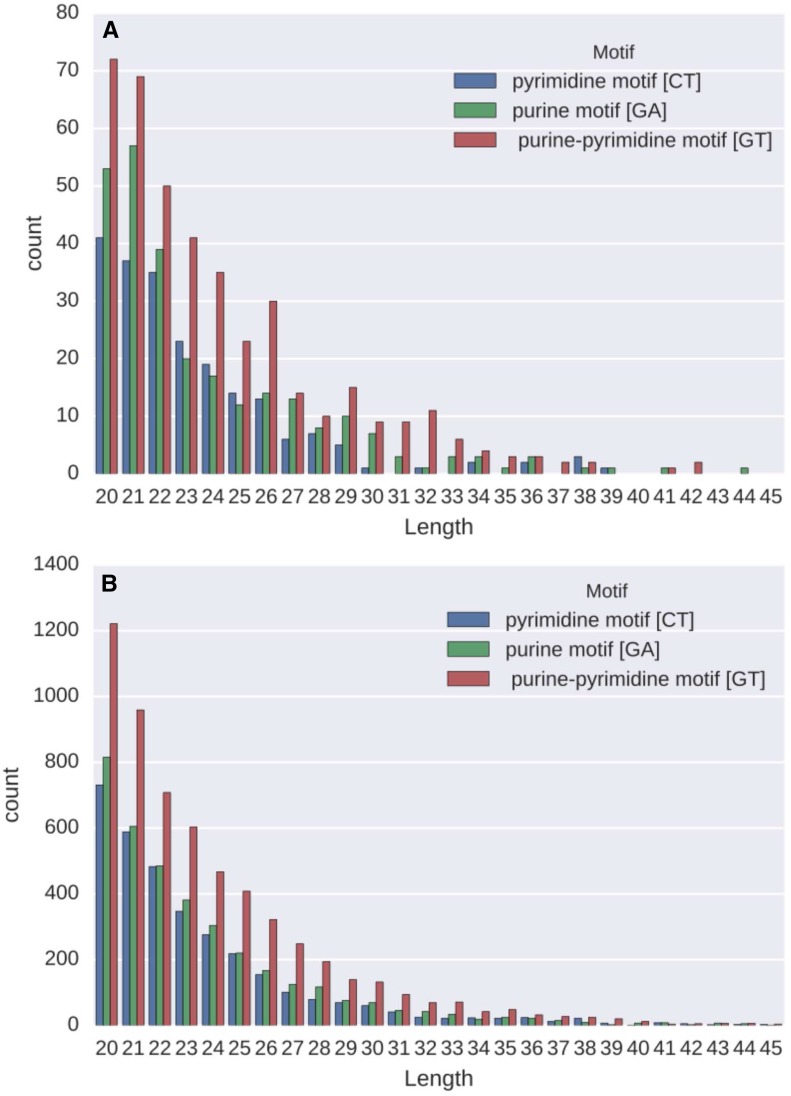
Distribution of TFOs by length and type. Distribution of TFOs of length <46 identified in lncRNAs. Bars show, for each length, the number of TFOs corresponding to the different motifs. (A) Distribution of the TFOs identified in lncRNAs (TFOs longer than 45 bases are listed in Table S2 in File S4). (B) Distribution of the TFOs of lengths <46 identified in pre-mRNAs (TFOs longer than 100 bases are listed in Table S3 in File S4).

The pre-lncRNAs contain 896 TFOs of lengths ranging from 20 to 58 ribonucleotides with a mean of 24 (the distribution of TFOs shorter than 46 bases is shown in Figure 1A). Only seven sequences with lengths over 45 bases have been identified in seven pre-lncRNAs (Table S2 in File S4). All the genes encoding these lncRNAs have unknown functions.

Pre-mRNAs containing 12,898 TFOs with an average length of 24 bases but with longer motifs, up to 460 ribonucleotides in length, were identified (the distribution of TFOs shorter than 46 bases is shown in Figure 1B, while TFOs longer than 100 bases are listed in Table S3 in File S4).

Detailed analysis showed that the TFOs are mainly located inside introns, with a proportion of 70% for both pre-lncRNAs and pre-mRNAs (Table 1).

To verify that most TFOs are not located in regions that might not be accessible for RNA:DNA triplex formation, we determined a rough estimate of the number of TFOs potentially included in the paired double helix (see *Materials and Methods*). At the most, the number of potentially not accessible TFOs does not exceed 25%.

A search for DNA sequences theoretically able to bind a third strand identified 9702 unique triplex-target sites (TTS) along the genome. Some sites are extensive, with a length of up to 490 nucleotides (the list of TTSs that are longer than 100 bases is presented in Table S4 in File S4, while the distribution of TTSs shorter than 51 bases is presented in Figure S1 in File S4). Detailed analysis revealed that 7486 TTSs (60% of the total number of sites) are located inside open reading frames (ORFs) with 5801 of them (77%) located inside introns (TTS column in Table 2). Many TTSs consist of repeated sequences along the chromosomes, strongly suggesting that a particular TFO might target many sites far beyond the gene that generates it.

The number of TTSs by gene ranges from 1 to 93 (Table S5 in File S4 lists the genes that contain 30 TTSs or more). Consequently, 2471 different genes contain triplex-target sites.

An assessment of potential triplex sites was performed by looking at the perfect matches between the TFOs and TTSs. Although the number of identified TFOs and TSSs is not very large (∼14,000 TFOs and <10,000 TSSs), the number of potential TFO:TSS matches is considerable. As an example, the 12,898 TFOs found in the mRNA genes can bind the 9702 TTSs identified on the genome in over 20 million different ways. In addition, due to their sequences being composed of repetitive patterns, each TFO can often target a TTS at numerous sites. To reduce this enumeration bias, we considered that a set of potential triplexes that overlapped over >10 bases constituted the same site. Despite this consideration, the combinatorics between the TFOs and TTSs still had a multiplier effect that was difficult to estimate. Discarding the matches that overlapped over >10 bases allowed a reduction in the number of potential triplexes by a factor of 5. However, exploring the matching of the triplex patterns found on the RNA sequences and the whole genome of *D. melanogaster* still revealed millions of potential combinations that can be formed along the genome.

**Figure 2:**
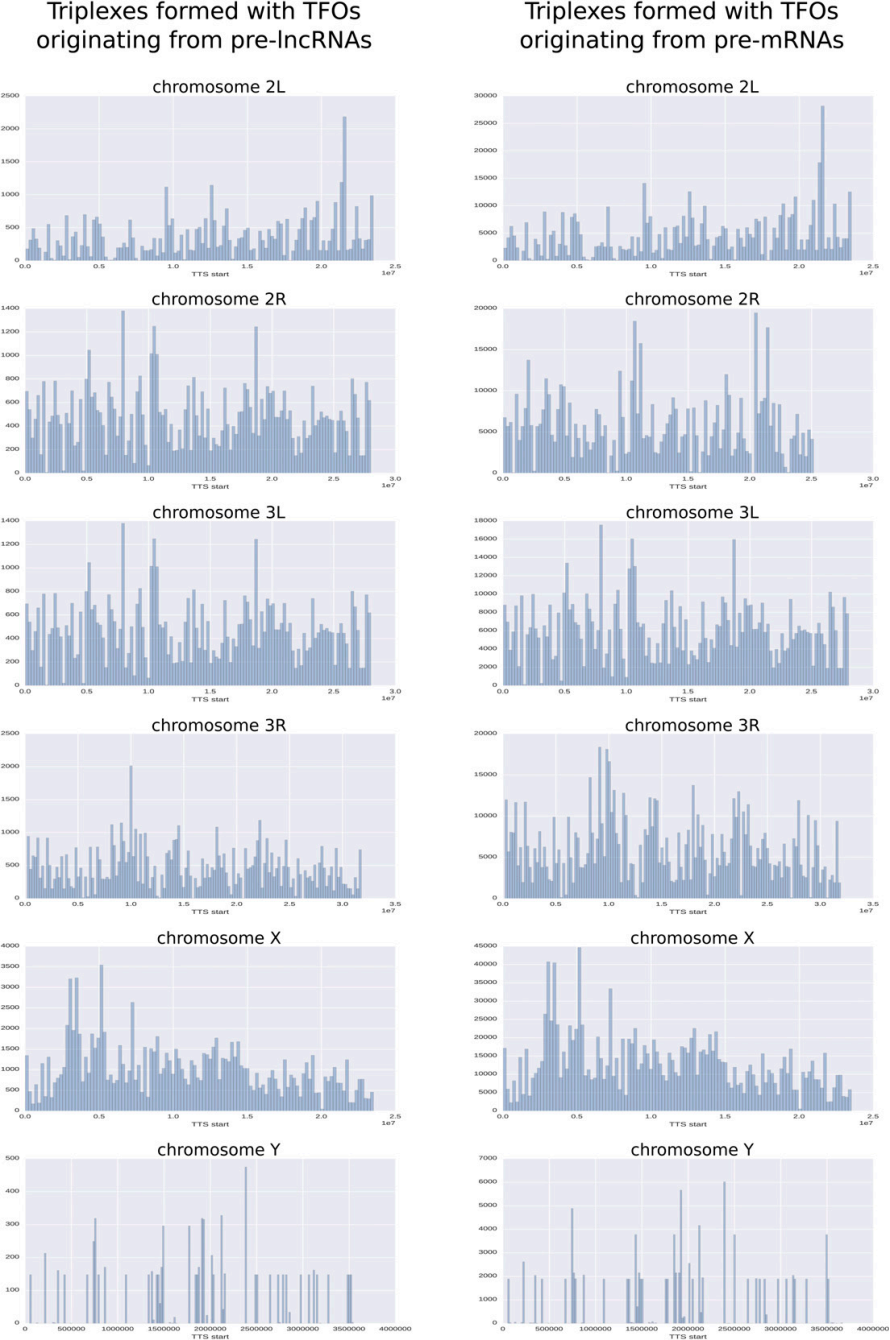
Distribution/localization of putative triplexes on chromosomes. Chromosomes were divided into bins of 200 kbp. Each bar on the plot represents the number of triplexes belonging to each bin. The histograms highlight some important hot spots that group a large number of triplexes. The most remarkable concentrations of triplexes occur on chromosome X at ∼5 Mbp, on chromosome 2L at ∼21 Mbp, and on chromosomes 3L and 2L at ∼10 Mbp.

In summary, 354,963 and 4,317,708 potential triplex sites have been found in the full genome for pre-lncRNA and pre-mRNA, respectively, and three fourths of them are localized inside genes (columns pre-lncRNA and pre-mRNA of Table 2). The localization of the triplex sites in introns massively overwhelms their presence in exons, which amounts to ∼10% for the two classes of RNA (Table 2). The distribution/localization of putative triplexes on the chromosomes is not a uniform distribution, as shown in Figure 2 and Table S6 in File S4. Chromosome X, for example, contains numerous potential triplexes: more than double those for other chromosomes of equivalent size. On each chromosome, we can see some important hot spots that group a large number of triplexes. The most remarkable concentration of triplexes occurs in positions that contain ∼21.5 million base pairs on chromosome 2L. This zone contains a cluster of histone genes (His1, His2B, His2A, His4, and His3). The putative triplexes are mainly located between His4 and His3. Several other concentrations of triplexes are detailed in Table S7 in File S4.

As we could expect, TFOs located in introns are the ones that contribute the most to the formation of triplexes. However, the localization on chromosomes of triplexes originating from introns and exons is very similar (Figures S2 and S3 in File S4).

Gene Ontology (GO) enrichment analysis of the mRNAs that mostly contribute to the formation of triplexes highlighted an overrepresentation of genes involved in receptor activity and molecular binding. These genes are also involved in a multitude of developmental processes, as revealed by the enrichment of corresponding annotations with p-values ranging from 10^−10^ to 10^−40^ (File S1, and Tables S8 and S9 in File S4 list the transcripts that contribute the most to the formation of triplexes, while the GO enrichment analyses are presented in File S2 and Tables S10 and S11 in File S4). GO enrichment analysis of the genes that are most targeted by lncRNA TFOs revealed an important overrepresentation of genes involved in molecular binding. Functions such as “histone binding,” “protein dimerization activity,” or “DNA binding” are enriched in the targets of both lncRNA TFOs and mRNA TFOs with incredibly low p-values (of the order of 10^−41^, 10^−24^, and 10^−17^, respectively, for the lncRNA TFO targets and 10^−17^, 10^−17^, and 10^−18^, respectively, for the mRNA TFO targets). Processes related to metabolism and cell organization are strongly emphasized in genes targeted by lncRNA TFOs with p-value scores exceeding 10^−30^ for “chromatin assembly,” “DNA packaging,” and “DNA-templated transcription.” The same biological processes are also overrepresented in genes targeted by mRNA TFOs but more modestly (with p-values still reaching 10^−10^). The specificity of genes targeted by mRNA TFOs over those targeted by lncRNA TFOs shows their important involvement in development and morphogenesis processes. A broad spectrum of processes, highlighted by very significant p-values, includes the following: “appendage development” (10^−29^), “axon development” (10^−20^), “cell differentiation” (10^−31^), “generation of neurons” (10^−29^), “metamorphosis” (10^−26^), “nervous system development” (10^−26^), “postembryonic development” (10^−26^), “tissue morphogenesis” (10^−32^), “tube morphogenesis” (10^−30^), and “axonogenesis” (10^−19^) (File S1 and Tables S12 and S13 in File S4 list the genes that are most targeted by TFOs, and GO enrichment analyses are summarized in File S2 and Tables S14–S16 in File S4). This high specific targeting strongly suggests that triplex DNA:RNA might constitute an unexpected layer of regulation in developmental biology.

**Figure 3:**
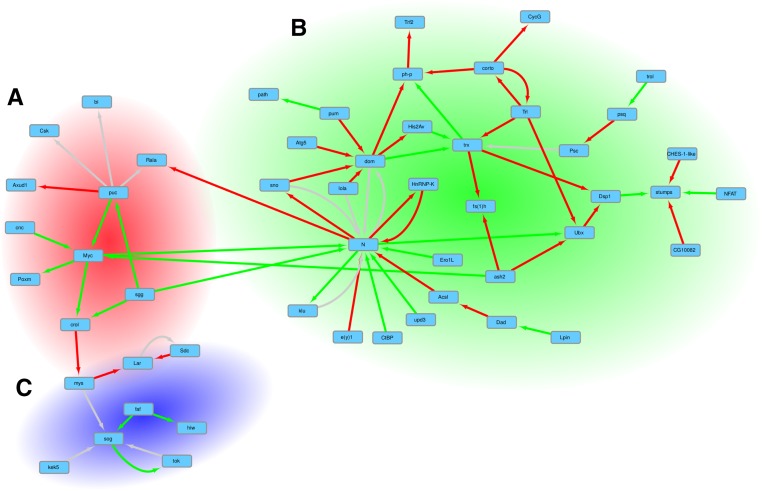
Network of genetic interactions between the genes that were most targeted by TFOs. The search for genetic interactions among the genes mostly targeted by pre-lncRNA and pre-mRNA TFOs identified a set of genes shaping a large interacting network. On the graph, red arrows represent a relation of suppression, green arrows represent a relation of enhancement, and gray arrows identify the cases when there is both a relation of suppression and a relation of enhancement. The graph can be divided into three subnetworks. The GO enrichment analysis performed on the genes composing the subnetworks produces an overrepresented annotation corresponding to different processes. (A) The 13 genes in this subnetwork are enriched with terms related to the regulation of cellular process, organ development, and morphogenesis. (B) This large cluster of 34 genes is enriched with terms related to morphogenesis and tissue development. (C) A cluster of eight genes related to axon guidance, locomotion, and the response to external stimulus.

The search for genetic interactions among the genes predictably targeted by lncRNA and mRNA TFOs identified a set of genes shaping a large interacting network (Figure 3). Not surprisingly, the GO enrichment analysis performed on this network highlighted annotations mainly related to development and morphogenesis (File S3). Interestingly, we distinguish three subnetworks corresponding to different biological processes very clearly on the graph of genes putatively targeted by lncRNA and mRNA TFOs (Figure 3 and File S3).

To support the bioinformatics data on a genome-wide scale, we attempted to address the question of a minimal biological validation that might corroborate the predictive analysis. We took advantage of thiazole orange-biotin (TO-biotin), which binds triplexes and quadruplexes with a higher affinity than DNA duplexes (Granzhan and Ihmels 2006; Lubitz *et al.* 2010; Ren and Chaires 2000), thus allowing us to perform pull-down experiments using the enzymatically cleaved genome of *Drosophila* under conditions that minimized duplex binding. Several runs of PCR amplification allowed us to sequence >300 colonies in which few sequences were highly represented. The results appear to show some variability between repeats with an evident affinity of TFO to streptavidin. The proportion of sequences corresponding to the triplex structure were constantly enriched compared to the ratio in the initial genomic fragments although they likely result from the combination TO/streptavidin isolation (93 compatible triplex structures that were at least 10 bases long and 24 that were at least 13 bases long originated from few genomic sequences). These results are even more significant if we accept 10% mismatches in the canonical patterns (100 with minimal 10-base lengths and 30 with minimal 20-base lengths) (see Table S18 in File S4). Optimizing and fine tuning the procedure with full genomic material is a reasonable goal due to the strong affinity of triplex made of synthetic oligonucleotides to the TO probe that has been reported in the literature (Granzhan and Ihmels 2006; Lubitz *et al.* 2010; Ren and Chaires 2000).

## Discussion

The properties of RNA, which are extensively documented in the literature, show their multi-functionality, such as binding specifically to proteins involved in chromatin remodeling and numerous regulatory transcriptional complexes (Elliot and Ladomery 2016). A less studied property is their capacity to engage in DNA:RNA triplex formation according to the Hoogsteen base pairing rules. We report that developmental genes are highly represented in the TTS, which corroborates the hypothesis of unexpectedly large-scale genome regulation mediated by the triplex DNA:RNA tertiary structure. In this regard, we investigated two classes of RNA, lncRNAs, and mRNA introns because their regulatory roles in gene expression have been extensively reported.

Many sequences of lncRNAs are derived from transposable elements, inactive pseudogenes, and intronic sequences and present few conserved features (Chen 2016; Goff and Rinn 2015; Li *et al.* 2016; Quinn and Chang 2015; Rinn and Chang 2012). lncRNAs operate through *trans*- and *cis*-acting functions that nucleate the assembly of RNA/DNA/proteins structures by recruiting proteins close to the site where they are transcribed (*cis*) or to remote loci (*trans*) (Chen 2016; Quinn and Chang 2015; Tsai *et al.* 2010). This mechanism explains lncRNA-mediated epigenetic control of cell fate through ordering the three-dimensional context of the nucleus. For instance, some lncRNAs operate sequentially in a temporal and spatial order to regulate homeobox (hox) genes (Mercer *et al.* 2009; Rinn and Chang 2012; Wilusz *et al.* 2009) by recruiting chromatin remodeling proteins that specify whether the zone is active or silent (Mercer *et al.* 2009; Rinn and Chang 2012; Wilusz *et al.* 2009). This is also the case for the lncRNA *Xist*, which is crucial for the epigenetic regulation observed in X chromosome inactivation (Mercer *et al.* 2009; Rinn and Chang 2012; Wilusz *et al.* 2009). Regarding the human DHFR gene, a lncRNA whose gene is located upstream of the DHFR promoter represses the expression DHFR. This inhibition acts by forming an RNA–DNA triplex structure with the DHFR promoter (Martianov *et al.* 2007). The modENCODE project (the consortium that built the encyclopedia of complete RNA elements on the genome scale available on the FlyBase public resource) has markedly highlighted the complex and unexpected dynamic of the spatial and temporal expression of the different RNA categories. Singularly, this finding has led to an unexpectedly large number of lncRNAs, although we are far from attributing biological functions to this newly emerged population of molecules.

Regarding the mRNA population, strong interest in intron functions has emerged over the past decade. A few examples of introns have shown induction of higher expression than that of the promoter itself for the same gene (Chorev and Carmel 2012; Juneau *et al.* 2006; Rose 2008; Shabalina *et al.* 2010). Many genes presenting intact coding sequences and promotors are not expressed without their main intron (Chorev and Carmel 2012; Juneau *et al.* 2006; Rose 2008; Shabalina *et al.* 2010). The transcript level in the intron-bearing gene can be boosted in a variable scale from 30 to a few 100 times more than the intronless counterpart (Chorev and Carmel 2012; Juneau *et al.* 2006; Rose 2008; Shabalina *et al.* 2010). It has also been reported that for many genes, the introns direct the tissue specificity of the expression pattern (Chorev and Carmel 2012; Juneau *et al.* 2006; Rose 2008; Shabalina *et al.* 2010). As expected, introns were shown to constitute repositories of *cis* elements, which participate in the regulation of transcription and genome organization. These *cis* elements include enhancers, silencers, or other elements that modulate the function of the upstream promoter. Interestingly, introns are also well known for hosting numerous lncRNAs.

More importantly, the dogma stipulates that the fragments of degraded cytosolic mRNA do not reenter by retro transfer into the nucleus, in contrast with the retention/accumulation of intronic RNAs in this organelle. However, most of the unspliced and flawed mRNAs are known to be trapped in the nucleus. These trapped transcripts likely include a considerable number of nascent transcripts, which leads to their concomitant *in situ* degradation (Gudipati *et al.* 2012; Houseley *et al.* 2006; Jackson *et al.* 2000; Kilchert *et al.* 2015). Therefore, the probable scenario is that the TFOs coming from exons of mRNA are produced *in situ* likely because of the blockage of the export of mature mRNAs to the cytoplasm resulting from quality check failure (Houseley *et al.* 2006). We observed that the introns appear to be the most important putative providers of the TFO, which makes sense due to their exclusive nuclear localization. The multi-subunit protein exosome complex that degrades aberrantly processed RNA generated by error in polymerase pausing, inefficient or erroneous splicing, a deficit in 5′ capping, and failure to add the poly(A) tail should provide a large pool of fragments with TFO motifs. These TFO motifs might have functional epigenetic roles by promoting TFO/TTS association and subsequent nucleation of DNA/protein complexes.

The presented genome-wide scale predictive analysis of triplex DNA:RNA according to Hoogsteen rules has amazingly shown that (i) 10% of the lncRNA and mRNA populations present perfect TFOs, (ii) the number of perfect TTSs on the genome is at an unexpectedly high level, and (iii) the triplexes appear to be predominantly localized to the introns of genes compared to other genome localizations. This finding strongly suggests that a few fragments of RNA might have broad actions on multiple loci on the chromosomes to realize a genome-wide scale of epigenetic control. We present theoretical evidence that a particular TFO in either lncRNA or mRNA might engage triplexes in thousands of sites embedded in the chromosomes. In this regard, we found that TFO-compatible motifs are mostly located between and/or outside the hairpin double strands in spliced lncRNAs. These TFOs could anchor lncRNAs at specific genomic sites and nucleate a higher order architecture of ribonucleoproteins to these locations. Furthermore, the introns of lncRNAs and mRNAs and the exons of mRNAs show equally low proportions of TFOs located in hairpins. Our data reveal a new approach for what is likely a new layer of genetic regulation by fragments of RNAs that have the potential to form triplex structures. This finding leads to the paradigm that some genes, through the expression of intronic RNAs and relevant lncRNAs, might potentially regulate, from a distance, numerous topologically distinct loci through DNA:RNA triplexes. The still poorly studied specific binding of a growing number of proteins to triplex DNA:RNA appears to be an unappreciated layer of regulation of gene expression that confers heritability (Matzke and Mosher 2014). We reasoned that chromosomes “bathe” in a soup of RNAs coming indistinctly from transfer, ribosomal, micro, lnc, and messenger RNAs along with their multiple products of degradation. We postulate that some of these molecules conforming to the Hoogsteen rules might have unknown and powerful structuring effects on the genome. To date, nothing is known or little is studied regarding placing the triplex structure in the context of developmental biology, the emergence of phenotypes, and the epigenetic variability of full organisms.

Our data led to the astonishing observation that triplexes match with very significant p-values to genes of the morphogenesis process. The large number of TTSs in development- and morphogenesis-related genes and their overwhelming selective matches with TFOs that originated from introns and lncRNAs strongly suggest that refined and poorly known regulation of expression operates through these tertiary structures. The discovery that a particular TFO in a lncRNA or intron can theoretically associate with a large number of TTSs at different locations of the genome was amazingly completed with the finding that the putative targets are restrictively associated with development- and morphogenesis-related genes. This finding strengthens the hypothesis of a new layer of on/off regulation of genes that are tightly controlled in a temporal and spatial order.

As a presage, many publications have reported the potential use of oligomers to form an *in situ* triplex as a tool in cancer or other human pathologies (Hélène 1991; Maher *et al.* 1991; Schleifman *et al.* 2008). The use of sequencing technology and bioinformatics tools along with public resources such as FlyBase will allow rapid progress in the exploration of this genome-wide scale potentiality.

## Supplementary Material

Supplemental material is available online at www.g3journal.org/lookup/suppl/doi:10.1534/g3.117.042911/-/DC1.
