# Supplementary material for: The Mapping of Predicted Triplex DNA:RNA in the Drosophila Genome Reveals a Prominent Location in Development- and Morphogenesis-Related Genes
Source: G3 (Bethesda). 2017 May 17;7(7):2295–304. doi: 10.1534/g3.117.042911 (PMC5499136; doi:10.1534/g3.117.042911)
Supplement: Supplementary file 4 [file 2295FileS4.pdf]

The mapping of predicted triplex DNA:RNA in the *Drosophila* genome reveals a prominent location in development- and morphogenesis-related genes

Authors: Claude Pasquier[1]*, Sandra Agnel[2], Alain Robichon[2]

Affiliations:

[1]Université Côte d'Azur, CNRS, I3S, France.

[2]Université Côte d'Azur, INRA, CNRS, ISA, France

*Correspondence to: claude.pasquier@unice.fr.

Supplementary Materials

Figure S1: Distribution by length and strand of a panel of TTSs identified on chromosomes.

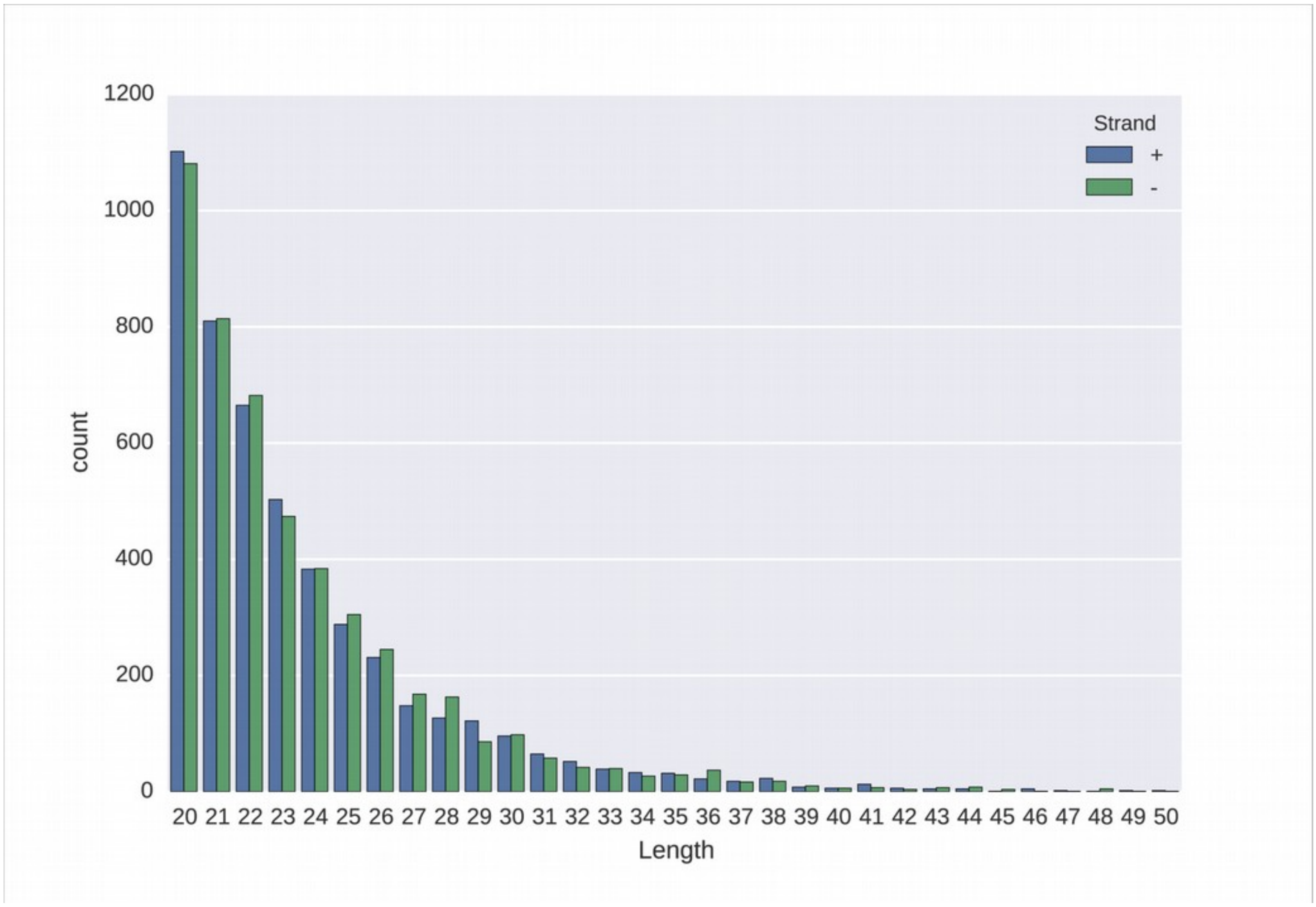

Distribution of TTSs shorter than 51 bases identified on chromosome. Bars show, for each length, the number of TTSs found on forward (+) and reverse (-) strands.

Figure S2: Distribution/localization of putative triplexes on chromosomes formed with TFOs originating from exons.

Triplexes formed with TFOs localized in lncRNAs' exons	Triplexes formed with TFOs localized in mRNAs' exons

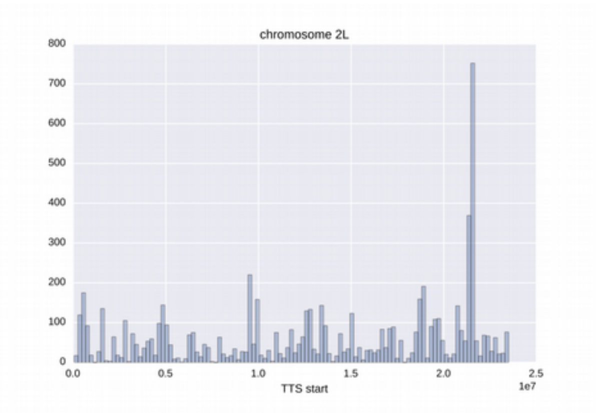

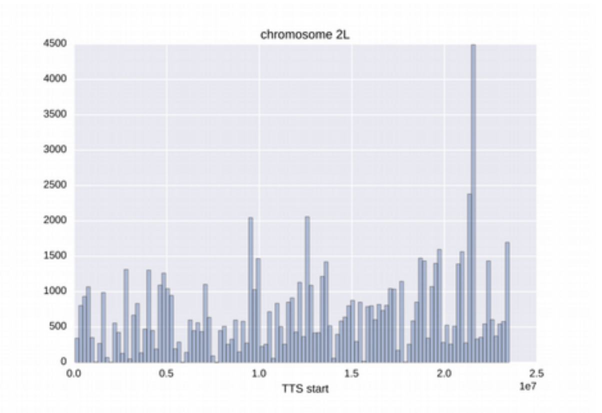

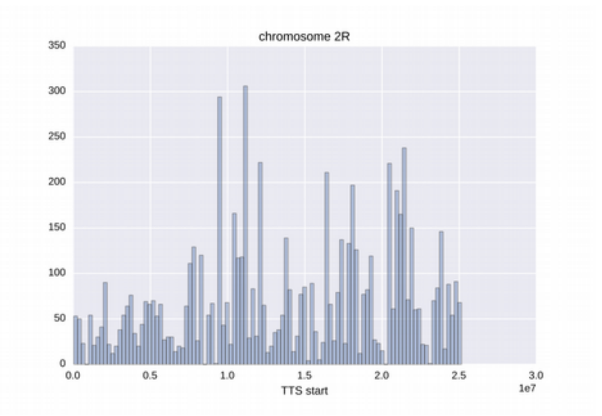

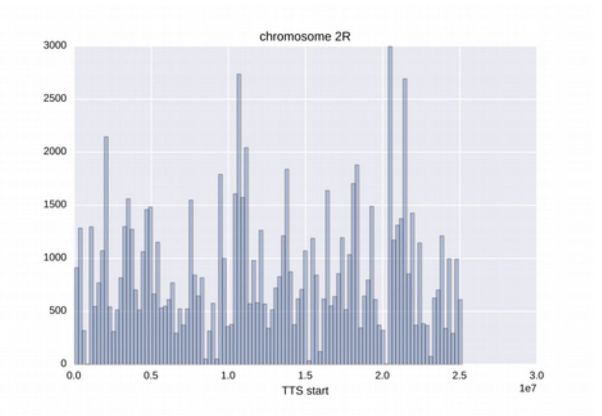

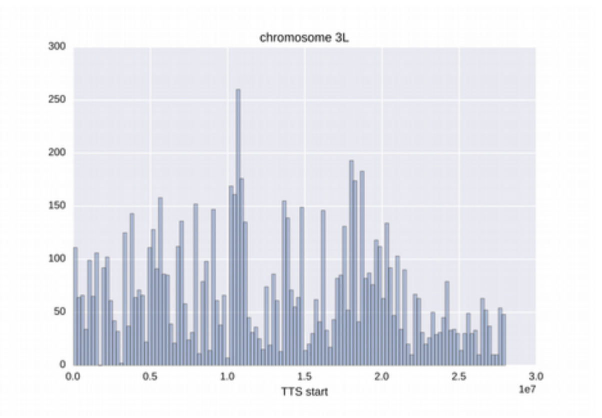

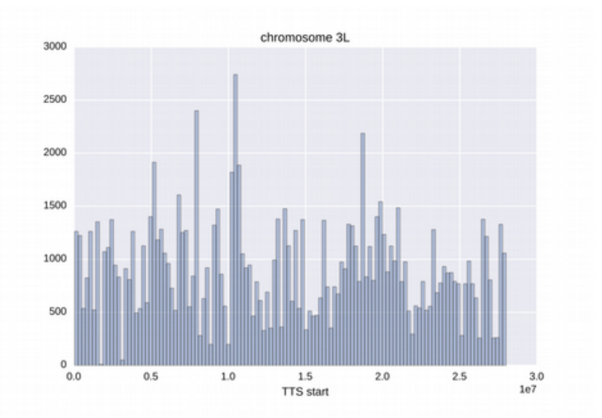

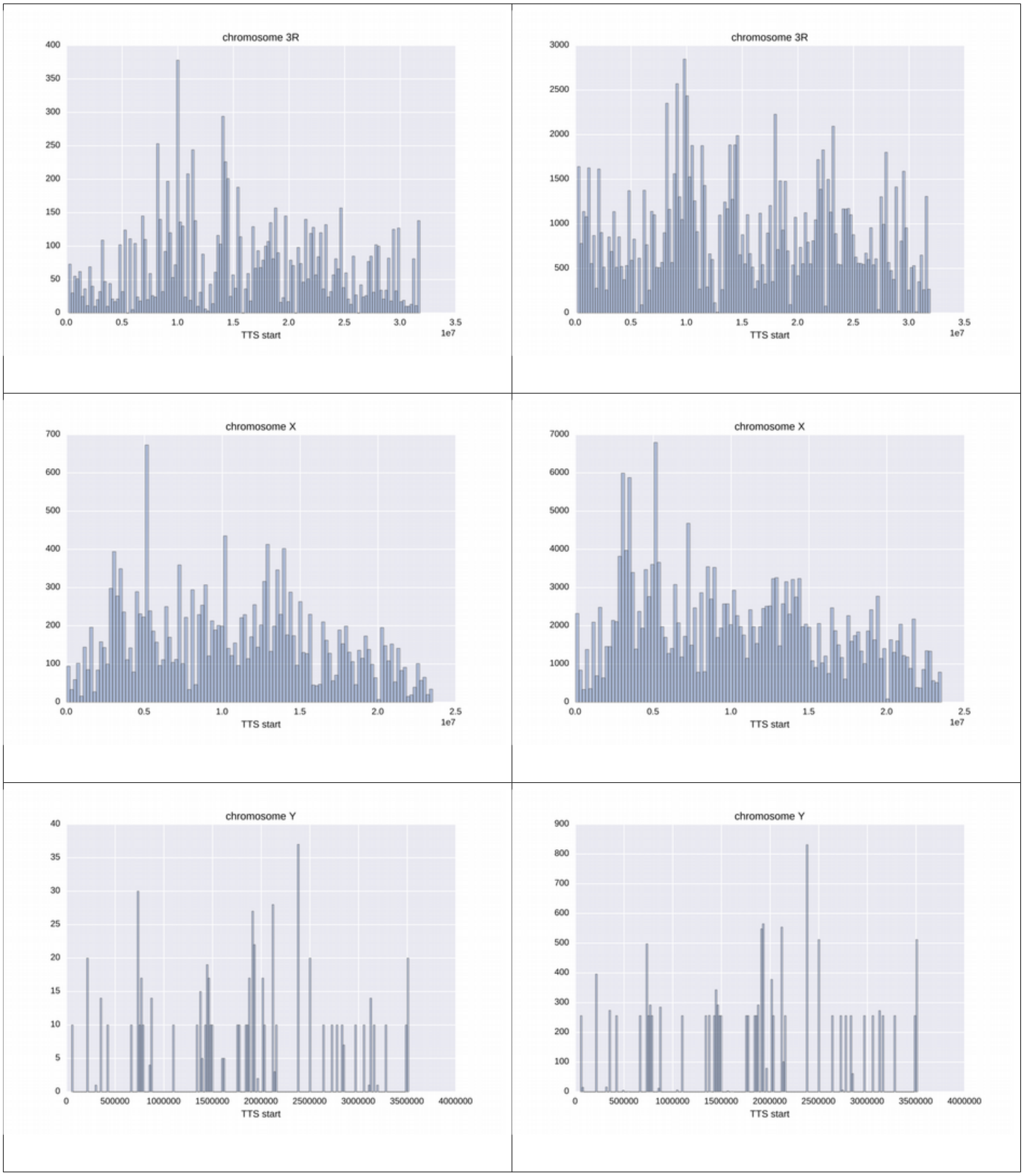

Chromosomes were divided into bins of 200kbp. Each bar on the plots represent the number of triplexes belonging to each bin.

Figure S3: Distribution/localization of putative triplexes on chromosomes formed with TFOs originating from introns.

Triplexes formed with TFOs localized in lncRNAs’ introns	Triplexes formed with TFOs localized in mRNAs’ introns
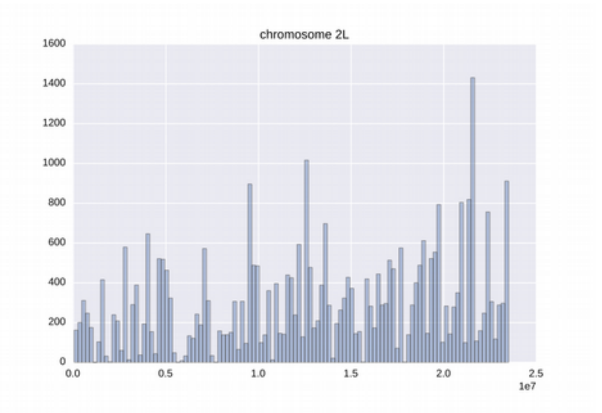	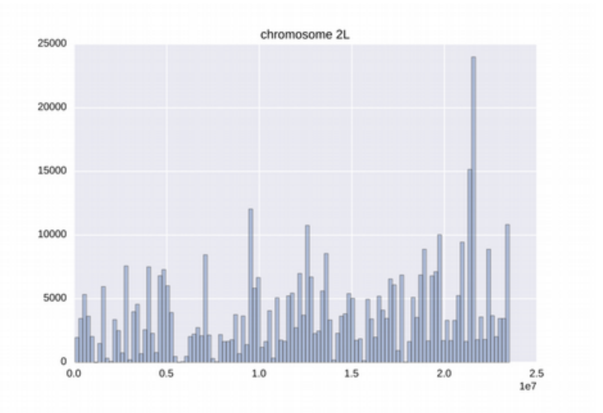
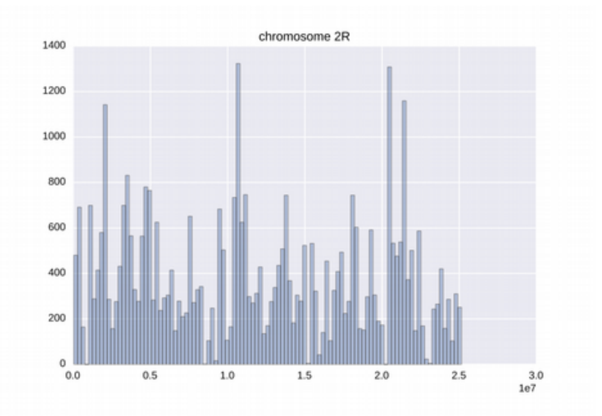	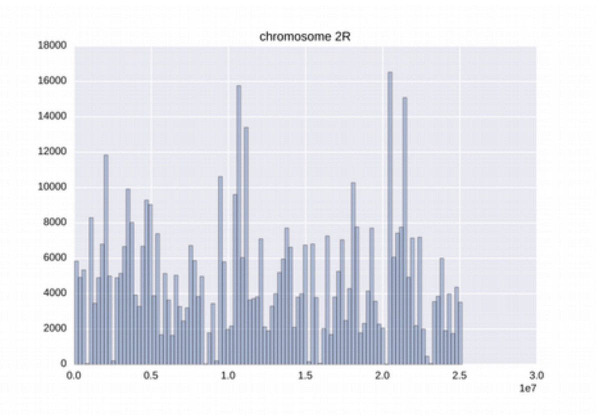
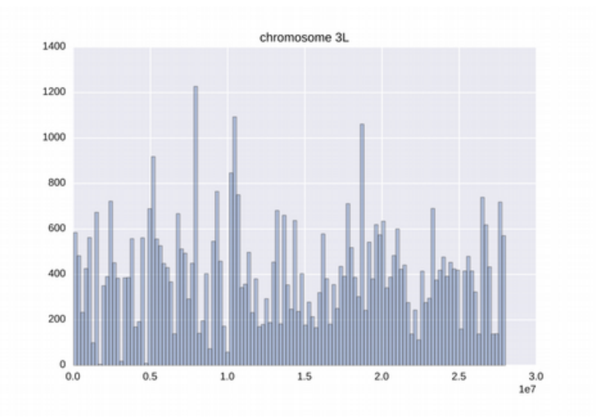	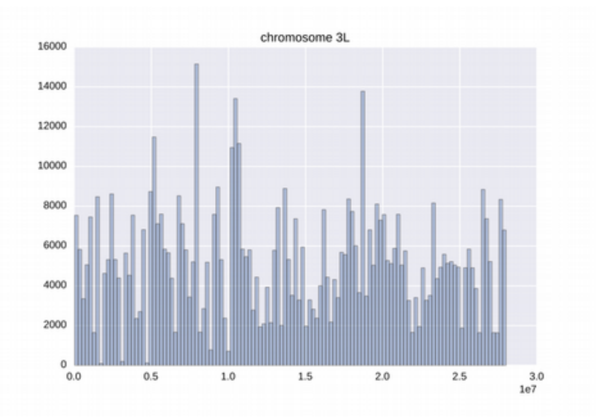

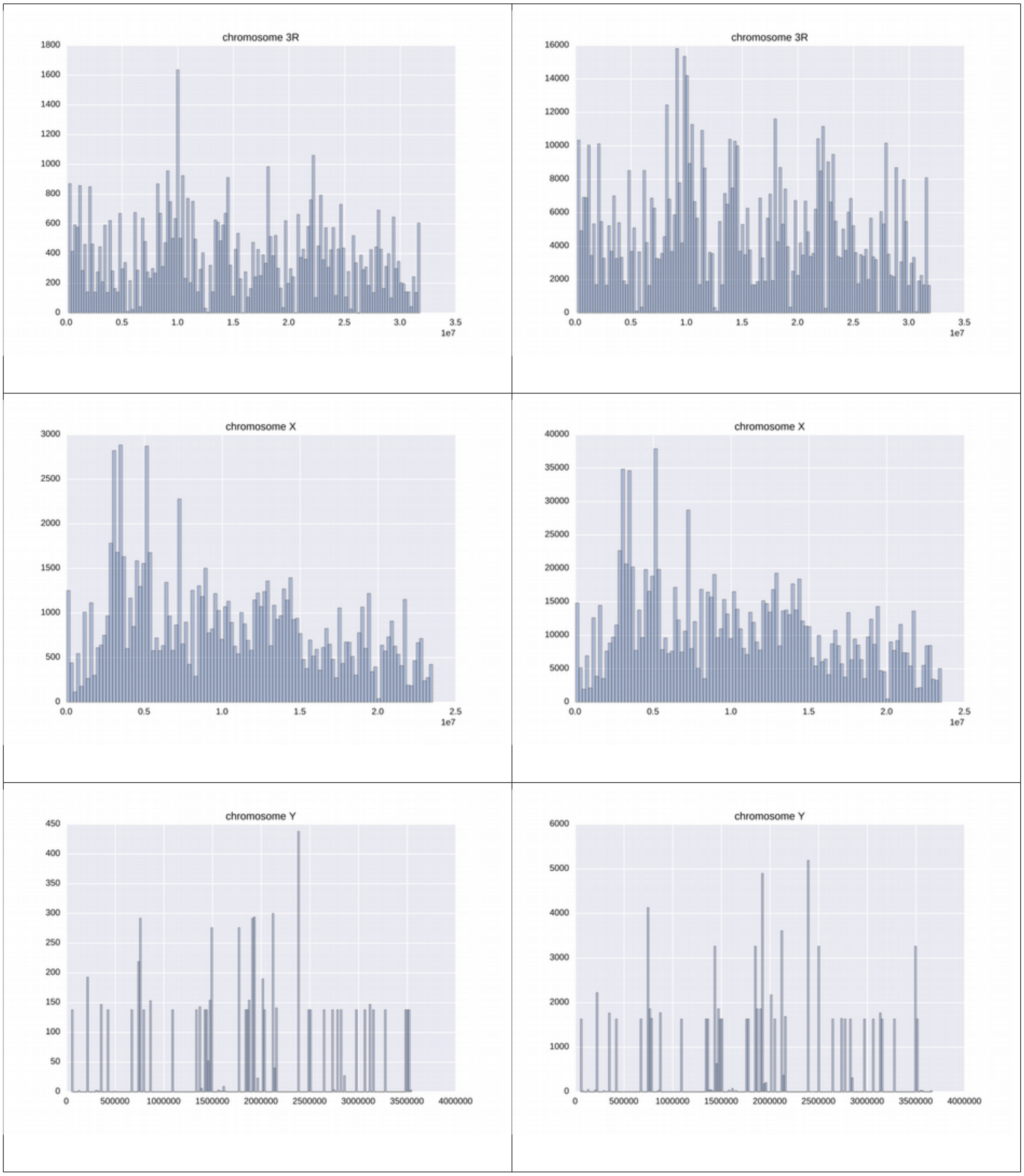

Chromosomes were divided into bins of 200kbp. Each bar on the plots represent the number of triplexes belonging to each bin.

Table S1: List of genes that include more than 50 TFOs.

Gene	Type	number of TFOs
FBgn0028369	mRNA	184
FBgn0267431	mRNA	153
FBgn0267001	mRNA	144
FBgn0029746	mRNA	118
FBgn0266098	mRNA	118
FBgn0265457	ncRNA	107
FBgn0052773	ncRNA	99
FBgn0267033	mRNA	86
FBgn0000479	mRNA	83
FBgn0266350	ncRNA	78
FBgn0025390	mRNA	78
FBgn0261260	mRNA	62
FBgn0261549	mRNA	59
FBgn0266096	ncRNA	57
FBgn0264001	mRNA	57
FBgn0259242	mRNA	56
FBgn0031016	mRNA	55
FBgn0003165	mRNA	53
FBgn0003380	mRNA	53
FBgn0029939	mRNA	51

269 pre-lncRNAs and 2888 pre-mRNAs contain triplex-forming oligonucleotides patterns (TFO). Most of them include only one TFO but several sequences contain more. The table lists the name and type of the 19 pre-RNA that include more than 50 TFOs.

Table S2: List of TFOs with a minimum length of 46 bases identified in pre-lncRNAs.

lncRNA	TFO start	TFO end	TFO length	Motif
FBgn0261700	46	92	46	purine
FBgn0263659	4773	4821	48	purine-pyrimidine
FBgn0264675	46826	46883	57	purine
FBgn0265161	590	639	49	purine-pyrimidine
FBgn0265457	95158	95209	51	purine-pyrimidine
FBgn0266005	27019	27067	48	purine-pyrimidine
FBgn0266199	33597	33655	58	pyrimidine motif

Pre-lncRNAs contain 896 TFOs of length ranging from 20 to 58 ribonucleotides. The table lists the TFOs longer than 40 bases. Columns include the name of the gene including the TFO, the start and end position of the TFO, its length and its motif.

Table S3: List of TFOs of length > 100 bases identified in pre-mRNAs.

mRNA	TFO start	TFO end	TFO length	Motif
FBgn0046323	588	894	310	pyrimidine
FBgn0046697	5561	6010	450	pyrimidine
FBgn0264561	8871	9007	140	purine-pyrimidine
FBgn0267430	93209	93568	360	purine
FBgn0267430	229808	229976	170	purine
FBgn0267430	229977	230440	460	purine
FBgn0267433	4858	5041	180	pyrimidine
FBgn0267433	12744	13150	410	pyrimidine
FBgn0031273	878	1082	200	purine
FBgn0031273	1146	1270	120	purine
FBgn0031273	1369	1516	150	purine
FBgn0031273	1517	1684	170	purine
FBgn0031273	1692	1907	220	purine
FBgn0031273	1908	2015	110	purine
FBgn0031273	2016	2137	120	purine

12898 TFOs, of length ranging from 20 to 460 bases were identified in pre-mRNAs. The table lists the TFOs longer than 100 bases. Columns include the name of the gene including the TFO, the start and end position of the TFO, its length and its motif.

Table S4: List of TTSs longer than 100 bases identified on chromosomes.

Chromosome	TTS start	TTS end	TTS length	TTS strand
**2L**	627559	627666	110	+
**2L**	23513493	23513604	110	+
**2L**	626797	626921	120	+
**2L**	627667	627788	120	+
**2L**	627020	627167	150	+
**2L**	23512816	23512967	150	+
**2L**	627168	627335	170	+
**2L**	626529	626733	200	+
**2L**	627343	627558	220	+
**2L**	23513038	23513331	290	+
**2R**	870230	870333	100	+
**2R**	748748	748931	180	+
**2R**	1826283	1826691	410	-
**2R**	1825696	1826183	490	-
**3R**	3033606	3033774	170	-
**3R**	3170014	3170373	360	-
**3R**	3033142	3033605	460	-
**Y**	1416903	1417060	160	-
**Y**	132445	132628	180	+
**Y**	200694	200877	180	+
**Y**	203437	203742	300	+
**Y**	3562332	3562638	310	-
**Y**	860181	860547	370	+
**Y**	124336	124742	410	+
**Y**	1642134	1642583	450	-

A search for sequences able to accommodate a third strand identified 9702 triplex-target sites (TTS) along the genome with a length of up to 490 nucleotides. The table lists the TTSs longer than 100 bases. Columns include the name of the chromosome where the TTS is located, the start and end position of the TTS, its length and its strand.

Table S5: List of genes that include at least 30 TTSs.

Gene	Protein coding gene?	number of TSSs
FBgn0028369	Yes	93
FBgn0267431	Yes	79
FBgn0266098	Yes	70
FBgn0029746	Yes	70
FBgn0267001	Yes	65
FBgn0267033	Yes	54
FBgn0265457	No	54
FBgn0000479	Yes	49
FBgn0052773	No	47
FBgn0266350	No	42
FBgn0261260	Yes	33
FBgn0261549	Yes	32
FBgn0000179	Yes	30
FBgn0266096	No	30

The number of TTSs by gene range from 1 to 93. The table lists the genes that contain 30 TTSs or more. Columns include the Id of the gene, its type (coding/non coding) and the number of TTSs identified in the ORF of the gene.

Table S6: Number of putative triplexes by chromosome depending of the origin of TFOs.

Chromosome	Number of putative triplexes obtained from pre-lncRNAs	Number of putative triplexes obtained from pre-mRNAs
X	115359	1465700
2L	41251	530002
2R	47444	607048
3L	58780	749403
3R	65829	841142
Y	7929	100075
4	1921	24338

Each chromosome is associated with the numbers of putative triplexes that are obtained by binding its TTSs and the TFOs originating from pre-lncRNAs and pre-mRNAs.

Table S7: Detail of genes localized near major concentrations of triplexes.

Chr.	Region of triplexes high density	Gene name	Gene type	Molecular function	Comment
X	5,085,000..5,255,000	rugose	coding	protein kinase A binding	
2L	21,410,000..21,540,000	His1<br>His2B<br>His2A<br>His4<br>His3	coding<br>coding<br>coding<br>coding<br>coding	DNA binding	This set of genes is reapeated ~20 times in the region. For each group of genes, the triplex concentrations are between His4 and His3.
3L	10,323,000..10,368,000	CR46006	non coding		
3L	10,772,000..10,826,000	CG43245<br>CR44544<br>CR44545<br>CR44546<br>CR46230<br>CR46231<br>CG12523<br>CR44347<br>CG42831<br>CR45247<br>CR45246	coding<br>non coding<br>non coding<br>non coding<br>non coding<br>non coding<br>coding<br>non coding<br>coding<br>non coding<br>non coding		A number of triplexes are formed in this area outside the ORF of genes

The distribution/localization of putative triplexes on chromosomes is not uniformly distributed. Each chromosome contains important hot spots that group a large number of triplexes. The table details 4 of the most remarkable concentrations of triplexes. Each region with a high density of triplexes, was scanned to find the genes included in the zone. The names of the genes, their types and their molecular function are specified.

Table S8: List of pre-lncRNAs that contribute the most to the formation of triplexes.

lncRNA	number of TSS matches
FBgn0266350	36280
FBgn0052773	34733
FBgn0265457	32808
FBgn0266096	15103
FBgn0267704	14789
FBgn0266180	9659
FBgn0264384	9144
FBgn0264675	7288
FBgn0266199	7284
FBgn0264678	5508
FBgn0267668	5452
FBgn0266348	4838
FBgn0266214	4186
FBgn0266376	3350
FBgn0051781	3345
FBgn0265663	3260
FBgn0266046	3189
FBgn0047205	3114
FBgn0266886	3114
FBgn0266005	2128
FBgn0267601	2026
FBgn0266313	1895
FBgn0263659	1819
FBgn0051386	1626
FBgn0266751	1106
FBgn0266851	1085
FBgn0267307	1038
FBgn0264842	1038
FBgn0262741	1038
FBgn0267229	1032

192 pre-lncRNAs contain TFOs that bind TTSs identified on the genome. The table lists the 30 lncRNAs that bind the largest number of TTSs.

Table S9: List of pre-mRNAs that contribute the most to the formation of triplexes.

mRNAs	number of TSS matches
FBgn0028369	74500
FBgn0267431	64820
FBgn0267001	48448
FBgn0266098	39466
FBgn0029746	39466
FBgn0259242	24314
FBgn0029939	24290
FBgn0267336	20069
FBgn0263780	20053
FBgn0000479	19992
FBgn0003165	19867
FBgn0000464	19004
FBgn0025390	18756
FBgn0052423	17684
FBgn0052791	17661
FBgn0267964	16857
FBgn0085387	16519
FBgn0085443	16212
FBgn0052600	16134
FBgn0267430	16106
FBgn0000179	15871
FBgn0264001	15102
FBgn0051163	14883
FBgn0040765	14840
FBgn0029657	14748
FBgn0031016	14178
FBgn0266429	13661
FBgn0001235	13044
FBgn0000114	13024
FBgn0267429	12883

2255 pre-mRNAs contain TFOs that bind TTSs identified on the genome. The table lists the 30 mRNAs that bind the largest number of TTSs.

Table S10: Enrichment analysis with GO Biological Process of pre-mRNAs that mostly contributes to the formation of triplexes.

**GO Biological process**	**P-value**
animal organ morphogenesis (GO:0009887)	3.75E-40
anatomical structure morphogenesis (GO:0009653)	1.32E-37
single-multicellular organism process (GO:0044707)	1.99E-36
biological regulation (GO:0065007)	2.17E-36
system development (GO:0048731)	1.84E-35
regulation of cellular process (GO:0050794)	4.16E-35
animal organ development (GO:0048513)	5.89E-35
regulation of biological process (GO:0050789)	6.36E-35
appendage morphogenesis (GO:0035107)	4.28E-33
post-embryonic appendage morphogenesis (GO:0035120)	7.88E-33
appendage development (GO:0048736)	1.21E-32
tissue morphogenesis (GO:0048729)	1.81E-32
imaginal disc-derived appendage morphogenesis (GO:0035114)	2.25E-32
morphogenesis of an epithelium (GO:0002009)	2.56E-32
post-embryonic animal organ development (GO:0048569)	3.48E-32
imaginal disc-derived appendage development (GO:0048737)	4.69E-32
post-embryonic animal organ morphogenesis (GO:0048563)	1.88E-31
imaginal disc morphogenesis (GO:0007560)	1.88E-31
epithelial tube morphogenesis (GO:0060562)	5.64E-30
single-organism process (GO:0044699)	7.88E-30
multicellular organismal process (GO:0032501)	1.73E-29
multicellular organism development (GO:0007275)	1.77E-29
tube development (GO:0035295)	2.19E-29
imaginal disc development (GO:0007444)	2.71E-29
developmental process (GO:0032502)	3.12E-29
tube morphogenesis (GO:0035239)	3.38E-29
single-organism developmental process (GO:0044767)	3.88E-29
epithelium development (GO:0060429)	6.36E-29
instar larval or pupal morphogenesis (GO:0048707)	8.93E-29
anatomical structure development (GO:0048856)	9.37E-29
tissue development (GO:0009888)	1.51E-28
metamorphosis (GO:0007552)	2.20E-28
post-embryonic development (GO:0009791)	2.79E-28
post-embryonic animal morphogenesis (GO:0009886)	3.37E-28
instar larval or pupal development (GO:0002165)	1.77E-26
imaginal disc-derived wing morphogenesis (GO:0007476)	4.95E-26

A limited number of pre-mRNAs contribute to the formation of a majority of triplexes. We selected, for Gene Ontology enrichment analysis with Biological Process, 381 transcripts that contributes to the formation of more than 75% of triplexes with a density of TFO-TSS matches above average. The table lists only annotations obtained with a p-value < 1E-25.

Table S11: Enrichment analysis with GO Molecular Functions of pre-mRNAs that mostly contributes to the formation of triplexes.

**GO Molecular Function**	**P-value**
protein binding (GO:0005515)	7.31E-09
binding (GO:0005488)	1.36E-08
signal transducer activity (GO:0004871)	9.78E-08
receptor activity (GO:0004872)	4.44E-07
molecular transducer activity (GO:0060089)	4.44E-07
signaling receptor activity (GO:0038023)	2.12E-06
nucleic acid binding transcription factor activity (GO:0001071)	2.22E-04
transcription factor activity	2.22E-04
sequence-specific DNA binding (GO:0043565)	1.68E-03
cyclic-nucleotide phosphodiesterase activity (GO:0004112)	2.30E-03
transmembrane signaling receptor activity (GO:0004888)	6.13E-03
transmembrane receptor activity (GO:0099600)	1.05E-02
transcription regulatory region DNA binding (GO:0044212)	1.27E-02
regulatory region nucleic acid binding (GO:0001067)	1.39E-02
regulatory region DNA binding (GO:0000975)	1.39E-02
potassium ion transmembrane transporter activity (GO:0015079)	2.74E-02
peptide receptor activity (GO:0001653)	3.18E-02
G-protein coupled receptor activity (GO:0004930)	4.98E-02

A limited number of pre-mRNAs contribute to the formation of a majority of triplexes. We selected, for Gene Ontology enrichment analysis with Molecular Function, 381 transcripts that contributes to the formation of more than 75% of triplexes with a density of TFO-TSS matches above average. The table lists all annotations obtained with a p-value < 1E-01.

Table S12: List of genes the most targeted by pre-lncRNA TFOs.

Gene	Number of TFO matches
FBgn0028369	4871
FBgn0267431	4577
FBgn0029746	3114
FBgn0266098	3114
FBgn0267001	2570
FBgn0266350	2544
FBgn0052773	2524
FBgn0265457	2496
FBgn0000479	1904
FBgn0267336	1719
FBgn0266096	1718
FBgn0029939	1627
FBgn0003165	1444
FBgn0261260	1345
FBgn0052600	1302
FBgn0267033	1262
FBgn0000179	1254
FBgn0261549	1239
FBgn0259242	1237
FBgn0263780	1232
FBgn0000464	1230
FBgn0085387	1136
FBgn0025390	1135
FBgn0267964	1082
FBgn0040765	1044
FBgn0052423	1043
FBgn0051163	1040
FBgn0029657	1020
FBgn0031016	1014
FBgn0267429	968

1542 genes contain TTSs that are targeted by pre-lncRNA TFOs. The table lists the 30 genes the most targeted with the number of matches.

Table S13: list of genes the most targeted by pre-mRNA TFOs.

Gene	Number of TFO matches
FBgn0028369	61911
FBgn0267431	57945
FBgn0029746	39287
FBgn0266098	39287
FBgn0267001	32413
FBgn0266350	31828
FBgn0265457	31602
FBgn0052773	31415
FBgn0000479	24052
FBgn0267336	21933
FBgn0266096	21534
FBgn0029939	20644
FBgn0003165	18385
FBgn0261260	16823
FBgn0052600	16693
FBgn0267033	15689
FBgn0263780	15614
FBgn0000464	15602
FBgn0261549	15504
FBgn0259242	15455
FBgn0000179	15385
FBgn0085387	14702
FBgn0025390	14624
FBgn0267964	13714
FBgn0052423	13328
FBgn0040765	13175
FBgn0051163	12993
FBgn0029657	12726
FBgn0265416	12713
FBgn0031016	12472

1914 genes contain TTSs that are targeted by pre-mRNA TFOs. The table lists the 30 genes the most targeted with the number of matches .

Table S14: Enrichment analysis with GO Biological Process of the genes the most targeted by pre-lncRNA TFOs.

GO Biological process	P-value
nucleosome assembly (GO:0006334)	3.23E-37
nucleosome organization (GO:0034728)	1.52E-36
DNA-templated transcription	1.93E-36
chromatin assembly (GO:0031497)	1.93E-36
protein-DNA complex assembly (GO:0065004)	8.08E-36
protein-DNA complex subunit organization (GO:0071824)	7.40E-35
chromatin assembly or disassembly (GO:0006333)	4.57E-34
DNA packaging (GO:0006323)	6.90E-34
DNA conformation change (GO:0071103)	3.38E-33
nucleic acid-templated transcription (GO:0097659)	2.19E-29
transcription	2.19E-29
RNA biosynthetic process (GO:0032774)	3.17E-29
cellular macromolecular complex assembly (GO:0034622)	3.20E-28
macromolecular complex assembly (GO:0065003)	8.85E-27
macromolecular complex subunit organization (GO:0043933)	3.84E-26
chromatin organization (GO:0006325)	6.70E-24
nucleobase-containing compound biosynthetic process (GO:0034654)	7.32E-24
aromatic compound biosynthetic process (GO:0019438)	2.92E-22
heterocycle biosynthetic process (GO:0018130)	6.74E-22
organic cyclic compound biosynthetic process (GO:1901362)	2.95E-21
RNA metabolic process (GO:0016070)	8.26E-21
chromosome organization (GO:0051276)	4.37E-20
cellular component assembly (GO:0022607)	2.23E-19
cellular component biogenesis (GO:0044085)	1.85E-18
nucleic acid metabolic process (GO:0090304)	1.06E-17
cellular nitrogen compound biosynthetic process (GO:0044271)	1.20E-16
cellular macromolecule biosynthetic process (GO:0034645)	2.60E-16
gene expression (GO:0010467)	2.69E-16
macromolecule biosynthetic process (GO:0009059)	5.66E-16
nucleobase-containing compound metabolic process (GO:0006139)	1.55E-14
heterocycle metabolic process (GO:0046483)	6.81E-14
cellular aromatic compound metabolic process (GO:0006725)	1.56E-13
organic cyclic compound metabolic process (GO:1901360)	4.06E-13
cellular biosynthetic process (GO:0044249)	4.16E-12
organic substance biosynthetic process (GO:1901576)	6.05E-12
cellular nitrogen compound metabolic process (GO:0034641)	1.25E-11
biosynthetic process (GO:0009058)	1.41E-11

A limited number of genes concentrate most of the triplexes. We used, for Gene Ontology enrichment analysis, the 35 genes targeted by pre-lncRNA TFOs that account for 75% of the total number of triplexes and that group a count of TFO-TSS matches per 1000 bases above average. The table lists the most representative annotations up to a p-value of 1E-10).

Table S15: Enrichment analysis with GO Biological Process of the genes the most targeted by pre-mRNA TFOs.

**GO Biological process**	**P-value**
regulation of cellular process (GO:0050794)	1.14E-42
animal organ morphogenesis (GO:0009887)	8.00E-42
regulation of biological process (GO:0050789)	1.29E-41
single-multicellular organism process (GO:0044707)	3.22E-41
anatomical structure morphogenesis (GO:0009653)	5.45E-41
biological regulation (GO:0065007)	3.55E-40
system development (GO:0048731)	1.19E-39
animal organ development (GO:0048513)	2.79E-37
single-organism developmental process (GO:0044767)	7.92E-35
developmental process (GO:0032502)	2.79E-34
multicellular organism development (GO:0007275)	1.26E-33
tissue morphogenesis (GO:0048729)	4.02E-33
morphogenesis of an epithelium (GO:0002009)	4.64E-33
anatomical structure development (GO:0048856)	5.62E-32
cell differentiation (GO:0030154)	4.08E-31
epithelial tube morphogenesis (GO:0060562)	9.54E-31
tube morphogenesis (GO:0035239)	1.37E-30
post-embryonic animal organ development (GO:0048569)	1.66E-30
cellular developmental process (GO:0048869)	2.36E-30
multicellular organismal process (GO:0032501)	2.41E-30
appendage development (GO:0048736)	7.12E-30
single-organism process (GO:0044699)	1.04E-29
tube development (GO:0035295)	1.25E-29
post-embryonic animal organ morphogenesis (GO:0048563)	2.40E-29
imaginal disc morphogenesis (GO:0007560)	2.40E-29
generation of neurons (GO:0048699)	9.85E-29
appendage morphogenesis (GO:0035107)	1.21E-28
imaginal disc-derived appendage development (GO:0048737)	1.62E-28
post-embryonic appendage morphogenesis (GO:0035120)	1.92E-28
imaginal disc-derived appendage morphogenesis (GO:0035114)	5.32E-28
cell development (GO:0048468)	8.89E-28
epithelium development (GO:0060429)	2.21E-27
imaginal disc development (GO:0007444)	2.61E-27
instar larval or pupal morphogenesis (GO:0048707)	3.67E-27
post-embryonic development (GO:0009791)	1.10E-26
metamorphosis (GO:0007552)	1.11E-26
response to chemical (GO:0042221)	1.15E-26
post-embryonic animal morphogenesis (GO:0009886)	1.44E-26
behavior (GO:0007610)	2.81E-26
tissue development (GO:0009888)	3.83E-26
cellular component organization (GO:0016043)	4.93E-26

We used, for GO enrichment analysis, the 447 genes targeted by pre-mRNA TFOs that account for 75% of the total number of triplexes and that group a count of TFO-TSS matches per 1000 bases above average. The table lists the most representative annotations up to a p-value of 1E-25).

Table S16: Enrichment analysis with GO Molecular Function of the genes the most targeted by TFOs.

GO Molecular Function	P-value	
	Targets of pre-mRNA TFOs	Targets of pre-lncRNA TFOs
protein binding (GO:0005515)	5.57E-22	3.14E-10
binding (GO:0005488)	8.98E-19	1.57E-04
DNA binding (GO:0003677)	2.92E-18	6.91E-17
protein dimerization activity (GO:0046983)	1.36E-17	1.82E-24
histone binding (GO:0042393)	3.77E-17	2.84E-41
nucleic acid binding transcription factor activity (GO:0001071)	1.19E-12	
transcription factor activity	1.19E-12	
protein heterodimerization activity (GO:0046982)	1.55E-12	2.06E-28
nucleic acid binding (GO:0003676)	2.75E-12	2.68E-14
sequence-specific DNA binding (GO:0043565)	1.25E-11	
heterocyclic compound binding (GO:1901363)	3.71E-10	1.14E-11
organic cyclic compound binding (GO:0097159)	5.46E-10	1.33E-11
signal transducer activity (GO:0004871)	6.10E-08	
receptor activity (GO:0004872)	1.03E-06	
molecular transducer activity (GO:0060089)	1.03E-06	
signaling receptor activity (GO:0038023)	7.60E-06	
transcription regulatory region DNA binding (GO:0044212)	6.80E-04	
regulatory region nucleic acid binding (GO:0001067)	7.58E-04	
regulatory region DNA binding (GO:0000975)	7.58E-04	
protein homodimerization activity (GO:0042803)	1.74E-03	
passive transmembrane transporter activity (GO:0022803)	1.98E-03	
channel activity (GO:0015267)	1.98E-03	
Rho guanyl-nucleotide exchange factor activity (GO:0005089)	2.41E-03	
substrate-specific channel activity (GO:0022838)	2.84E-03	
identical protein binding (GO:0042802)	5.37E-03	
cation channel activity (GO:0005261)	7.12E-03	
RNA polymerase II transcription factor activity	9.05E-03	
ion channel activity (GO:0005216)	9.23E-03	
transmembrane signaling receptor activity (GO:0004888)	9.85E-03	

A limited number of genes concentrate most of the triplexes. We selected the genes targeted by TFOs that account for 75% of the total number of triplexes and that group a count of TFO-TSS matches per 1000 bases above average. This represents 35 genes targeted by pre-lncRNA TFOs and 447 genes targeted by pre-mRNA TFOs. Enrichment analyses with GO Molecular Function, performed on these two sets of genes, produced two lists of overrepresented annotations. The table lists the most representative annotations for genes targeted by pre-mRNA TFOs (up to a p-value of 1E-2) and displays the p-value scored by enrichment analysis of the list of genes targeted by pre-lncRNA TFOs. Empty cells in the column corresponding to pre-lncRNA TFOs indicate that the enrichment of the corresponding annotation is associated with a bad p-value > 1E-2.

Table S17: Distribution of TSSs and triplex matches on the genome with an allowed error rate of 5 %.

	TTS		lncRNA		mRNA	
	number	%	number	%	number	%
genome	40,086	100.00%	3,083,421	100.00%	38,759,372	100.00%
genes	30,568	76.25%	2,371,720	76.91%	29,716,660	76,66%
introns	22,719	56.67%	1,994,533	64,68%	25,408,195	65,55%
exons	7,849	19.58%	377,187	12,23%	4,308,465	11,11%

Table S18: Quantification of compatible triplex sequences found in the DNA pull down obtained with the triplex biotinyl probe (TO-biotin).

Matching method	Minimal length of triplex	Number of sequences
Perfect match	56	1
	24	2
	14	5
	13	24
	12	25
	11	30
	10	93
10% mismatches	93	1
	34	2
	22	3
	21	17
	20	30
	16	33
	15	56
	14	60
	13	63
	12	96
	11	100
	10	100

The DNA fragments recovered from the pull down obtained with TO biotin were analyzed for the presence of triplex compatible sequences. The minimal length for triplex structure is 9 bases. The analysis was performed with a minimal 10 bases length up to 56 for perfect triplex and 93 when 10% mistakes were included.

File S1: Complete versions of data listed in Tables S8-S9 and S12-S13.

File S2: Complete versions of data listed in Tables S10-S11 and complete GO annotations used to build Tables S14-S16.

File S3: Data associated with Figure 3.
List of genes on the graph, composition of each cluster, GO enrichments obtained for the whole graph and the 3 identified clusters.
